# Executive functioning and serum lipid fractions in Parkinson’s disease—a possible sex-effect: the PACOS study

**DOI:** 10.1007/s00702-022-02460-1

**Published:** 2022-01-13

**Authors:** Antonina Luca, Roberto Monastero, Calogero Edoardo Cicero, Roberta Baschi, Giulia Donzuso, Giovanni Mostile, Vincenzo Restivo, Lucia Di Giorgi, Maria Caccamo, Mario Zappia, Alessandra Nicoletti

**Affiliations:** 1 Department of Surgical and Medical Sciences Advanced Technologies “G.F. Ingrassia”, University of Catania, Via Santa Sofia 78, 95123 Catania, Italy; 2 Department of Biomedicine, Neuroscience and Advanced Diagnostics, University of Palermo, Palermo, Italy; 3 Department of Sciences for Health Promotion and Mother-Child Care, University of Palermo, Palermo, Italy

**Keywords:** Parkinson’s disease, Cholesterol, Triglycerides, Executive functions

## Abstract

The association between dyslipidemia and cognitive performance in Parkinson’s disease (PD) patients still needs to be clarified. Aim of the study was to evaluate the presence of possible associations between serum lipids fractions and executive dysfunction also exploring the sex-specific contribute of lipids level on cognition. Patients from the PACOS cohort, who underwent a complete serum lipid profile measures (total cholesterol-TC, low-density lipoprotein cholesterol-LDL, high-density lipoprotein cholesterol-HDL and triglycerides-TG) were selected. Adult Treatment Panel III guidelines of the National Cholesterol Education Program were used to classify normal/abnormal lipid fractions. Executive functioning was assessed with the Frontal Assessment Battery (FAB). Logistic regression was performed to assess associations between lipids fractions and FAB score. Correlations between lipids fractions and FAB score were explored. Sex-stratified analysis was performed. Three hundred and forty-eight PD patients (148 women; age 66.5 ± 9.5 years; disease duration 3.9 ± 4.9 years) were enrolled. Women presented significantly higher TC, LDL and HDL than men. In the whole sample, any association between lipid profile measures and FAB score was found. Among women, a positive association between hypertriglyceridemia and FAB score under cutoff was found (OR 3.4; 95%CI 1.29–9.03; *p* value 0.013). A statistically significant negative correlation was found between the FAB score and triglyceride serum levels (*r* = − 0.226; *p* value 0.005). Differently, among men, a statistically significant negative association between hypercholesterolemia and FAB score under cutoff (OR 0.4; 95%CI 0.17–0.84; *p *value 0.018) and between high LDL levels and FAB score under cutoff (OR 0.4; 95%CI 0.18–0.90; *p* value 0.027) were found. Our data suggest a sex-specific different role of lipids in executive functioning.

## Introduction

In Parkinson’s disease (PD) cognitive decline is mainly characterized by executive dysfunction, set of cognitive abilities including planning, monitoring, set-shifting, inhibiting behaviors and manipulating information (Fang et al. 2020).

Interestingly, an increasing literature evidence suggests relevant sex-differences in the clinical presentation of cognitive impairment in PD. Specifically, females seem to present a lower risk and slower progression of cognitive decline when compared to males (Iwaki et al. 2021; Nicoletti et al. 2017). To date, several modifiable risk factors have been associated with cognitive decline in PD patients, including systolic blood pressure, diabetes, smoking, decreased physical activity, obesity and dyslipidemia (Nicoletti et al. 2021; Guo et al. 2019).

Considering the devastating effects of cognitive impairment on the patient’s quality of life and their family caregivers’ well-being, the identification of possibly modifiable risk factors is undoubtedly useful. Concerning dyslipidemia, while several studies have investigated the possible link between altered serum lipid profile measures and cognitive decline both in patients with Alzheimer’s disease (Bernath et al. 2020; Sàiz-Vazquez et al. 2020) and in non-demented elderly (Parthasarathy et al. 2017), the role of serum lipids in PD is quite controversial. Although not entirely consistent, some studies suggested that subjects with high levels of cholesterol and low-density lipoproteins have a lower PD risk, thus supporting the role of statins as a risk factor for PD (Potashkin et al. 2020; Fu et al. 2020). To date only few studies have investigated the effects of dyslipidemia on cognitive performance in PD, reporting conflicting results (Choe et al. 2021; Huang et al. 2018; Mollenhauer et al. 2019; Bakeberg et al. 2021). Furthermore, most of the existing studies did not consider the sex-specific differences when evaluating possible associations between lipids levels and cognition.

This study, which is part of The PArkinson’s disease COgnitive impairment Study (PACOS) (Monastero et al. 2018; Nicoletti et al. 2019; Mostile et al. 2019; Baschi et al. 2019; Cicero et al. 2019; Luca et al. 2021; Donzuso et al. 2021), aimed at investigating the presence of possible associations between serum lipids fractions and executive dysfunction in patients suffering from PD, also exploring the sex-specific contribute of lipids level on cognition.

## Methods

### Study population

Patients affected by PD diagnosed according to the Brain Bank criteria (Gibb and Lees 1988), who attended the Neurologic Unit of the “Policlinico-San Marco” in Catania and the Memory and Parkinson’s disease Center of the “Policlinico Paolo Giaccone” in Palermo, were enrolled in the PACOS cohort, including 659 non-demented PD patients at baseline (Monastero et al. 2018). From the PACOS cohort, all PD patients (*n* = 348) who underwent a complete serum lipid profile assessment, including total cholesterol (TC), low-density lipoproteins (LDL), high-density lipoproteins (HDL) and triglycerides (TG) dosage during the same week of the neuropsychological evaluation were enrolled. Patients who did not complete the assessment of executive functioning were excluded from the analysis. All participants provided written informed consent prior to entering in the study, which was approved by the Local Ethical Committee and was in accordance with the Declaration of Helsinki.

### Clinical and neuropsychological evaluation

Patients underwent a comprehensive neurological and neuropsychological examination performed by movement disorders specialists. Demographic, clinical and pharmacological data were collected from the patient’s medical records. PD severity was evaluated in “off” state with the Unified Parkinson Disease Rating Scale-Motor Examination (UPDRS-ME). PD medications were converted in Levodopa Equivalent Daily Dosage (LED) (Tomlinson et al. 2010). The assessment of executive functioning was made with the administration of the Frontal Assessment Battery (FAB), considered useful tool for the screening of executive dysfunction in PD for its good discriminant and concurrent validities (Lima et al. 2008). Age- and education-adjusted norms for the Italian population were applied using a cutoff score of 13.5 (Appollonio et al. 2005).

### Serum analyses

Fasting blood samples were collected prior to clinical and psychological assessments during routine biochemistry diagnostic work-up. According to the National Cholesterol Education Program Adult Treatment Panel III (Adult Treatment Panel III 2001), the following cutoffs of serum lipid levels were adopted:

- Hypercholesterolemia: fasting TC level ≥ 200 mg/dl
- Hypertriglyceridemia: fasting TG level ≥ 150 mg/dl
- Low HDL cholesterol: fasting HDL level less than 40 mg/dl (men) or 50 mg/dl (women)
- High LDL cholesterol: fasting LDL level ≥ 130 mg/dl

### Statistical analysis

Data were analyzed using STATA 15 software packages (StataCorp, College Station, TX, United States). Data cleaning was performed before data analysis considering both range and consistence checks. Quantitative variables were described using mean and standard deviation. The difference between means and proportions was evaluated by the *t* test and the Chi square test, respectively. In case of a not-normal distribution, appropriate non-parametric tests were performed.

First, univariate analysis logistic regression was performed to evaluate possible associations between serum lipid profile and FAB score, considered as outcome variables. Subsequently, multivariate analysis was performed adjusting for age, sex, education, disease duration, and UPDRS-ME, considered a priori confounders. Serum lipid measures and FAB scores were analyzed as both categorial (presence/absence of hypertriglyceridemia, hypercholesterolemia, high LDL, high HDL and FAB score under cutoff) and continuous variable.

Presence of interaction between serum lipids fractions and sex was tested using the Likelihood Ratio Test comparing the log-likelihood of the model with and without the interaction parameter. A sex-stratified analysis was also performed.

Pearson’s correlation analysis was performed to investigate possible correlations between FAB and the specific serum lipid fractions. Linear regression was performed to adjust for age and disease duration. The significance level was set at 0.05 and the 95% confidence intervals (CI) were calculated.

## Results

Three hundred and forty-eight PD patients (148 women, 200 men) were enrolled. Demographic and clinical characteristics of the sample are shown in Table 1. At both univariate analysis and multivariate analysis, no statistically significant association was found between serum lipid fractions (TG, TC, LDL, HDL) and FAB score (Table 2). Finally, no statistically significant correlations were found between serum lipid fractions and FAB score.

**Table 1:** General characteristics of the sample

	Total (n.348)	Women (n.148)	Man (n.200)	OR	95%CI	*p* value
Age, yrs	66.5 ± 9.5	66.7 ± 9.2	66.5 ± 9.7	0.99	0.97–1.02	0.824
Education, yrs	8.5 ± 4.8	7.8 ± 4.8	9.0 ± 4.8	1.05	1.00–1.10	0.027
Age at PD onset, yrs	62.6 ± 10.5	61.9 ± 10.4	63.1 ± 10.6	1.01	0.99–1.03	0.317
Disease duration, yrs	3.9 ± 4.9	4.8 ± 5.0	3.4 ± 4.7	0.94	0.89–0.98	0.011
UPDRS-ME score	27.1 ± 12.9	28.2 ± 12.6	26.2 ± 13.2	0.98	0.97–1.00	0.159
LED mg/die	333.4 ± 347.2	350.1 ± 360.0	321.2 ± 337.9	0.99	0.99–1.00	0.443
MMSE score	27.0 ± 2.1	26.8 ± 2.4	27.3 ± 1.9	1.10	0.99–1.22	0.059
FAB score	14.1 ± 2.8	13.8 ± 3.1	14.3 ± 2.6	1.07	0.99–1.15	0.076
TG mg/dl	113.7 ± 61.6	109.6 ± 56.7	116.8 ± 65.0	1.00	0.99–1.00	0.278
TC mg/dl	188.2 ± 41.6	202.9 ± 43.2	177.3 ± 36.9	0.98	0.97–0.98	< 0.001
HDL mg/dl	50.6 ± 15.1	56.8 ± 15.3	45.9 ± 13.2	0.94	0.92–0.95	< 0.001
LDL mg/dl	115.6 ± 35.4	124.4 ± 38.9	109.0 ± 31.2	0.98	0.98–0.99	< 0.001

Data are expressed as mean ± standard deviation or number and percentage

*OR* odd ratio; *CI* confidence interval; *yrs* years; *PD* Parkinson’s disease; *UPDRS-ME* Unified Parkinson’s Disease Rating Scale-Motor Examination; *LED* levodopa equivalent dosage; *MMSE* mini mental state examination; *FAB* frontal assessment battery; *TG* triglycerides; *TC* total cholesterol; *HDL* high density lipoproteins; *LDL* low density lipoproteins

**Table 2:** Serum lipid fractions and FAB: univariate and multivariate analysis

			Univariate analysis			Multivariate analysis**		
	FAB ≥ 13.5 (n. 240)	FAB < 13.5 (n. 108)	OR	95%CI	*p* value	OR	95%CI	*p* value
Hypertriglyceridemia	38 (15.8)	21 (19.4)	1.3	0.71–2.31	0.407	1.1	0.58–2.20	0.700
Hypercholesterolemia	93 (38.7)	39 (36.1)	0.9	0.55–1.43	0.639	0.8	0.48–1.46	0.550
High LDL	83 (34.6)	34 (31.5)	0.9	0.53–1.41	0.571	0.9	0.52–1.57	0.734
Low HDL	87 (36.2)	40 (37.0)	1.0	0.64–1.65	0.888	0.9	0.56–1.58	0.819

	FAB ≥ 13.5 (n. 97)	FAB < 13.5 (n. 51)	Univariate analysis			Multivariate analysis**		
Women (n.148)			OR	95%CI	*p* value	OR	95%CI	*p* value
Hypertriglyceridemia	8 (8.2)	12 (23.5)	**3.4**	**1.29–9.03**	**0.013**	2.4	0.80–7.41	0.117
Hypercholesterolemia	46 (47.2)	30 (58.8)	1.6	0.79–3.14	0.189	1.5	0.69–3.48	0.283
High LDL	38 (39.2)	25 (49.0)	1.5	0.75–2.95	0.251	1.7	0.78–3.85	0.175
Low HDL	36 (37.1)	19 (37.2)	1.0	0.49–2.02	0.986	1.3	0.56–2.90	0.545

Men (n.200)	FAB ≥ 13.5 (n. 143)	FAB < 13.5 (n. 57)	OR	95%CI	*p* value	OR	95%CI	*p* value
Hypertriglyceridemia	30 (20.9)	9 (15.8)	0.7	0.31–1.60	0.405	0.7	0.28–1.70	0.425
Hypercholesterolemia	**49 (32.9)**	**9 (15.8)**	**0.4**	**0.17–0.84**	**0.018**	**0.4**	**0.15–0.93**	**0.035**
High LDL	**45 (31.5)**	**9 (15.8)**	**0.4**	**0.18–0.90**	**0.027**	0.4	0.16–1.04	0.061
LowHDL	51 (35.7)	21 (36.8)	1.0	0.55–1.99	0.876	0.8	0.37–1.58	0.470

Data are expressed as number and percentage

Bold values indicate statistically significance

*LDL* low-density lipoproteins, *HDL* high-density lipoproteins, *FAB* frontal assessment battery, *OR* odd ratio, *CI* confidence interval

*Adjusted for age, sex, education, disease duration, Unified Parkinson’s Disease Rating Scale-Motor Examination; **adjusted for age, education, disease duration, Unified Parkinson’s Disease Rating Scale-Motor Examination

### Sex-stratified analysis

At the logistic regression model, a significant interaction between sex and TG (OR 0.20; 95%CI 0.05–0.73; *p* value 0.015), TC (OR 0.24; 95%CI 0.08–0.68; *p* value 0.008) and LDL (OR 0.27; 95%CI 0.09–0.78; *p* value 0.015) but not HDL (OR 1.04; 95%CI 0.40–2.69; *p* value 0.926) was found and a stratified analysis by sex was performed.

Considering sex-differences, women were significantly less educated, had a longer disease duration and had significantly higher serum TC, HDL and LDL levels than men.

**Fig. 1:**
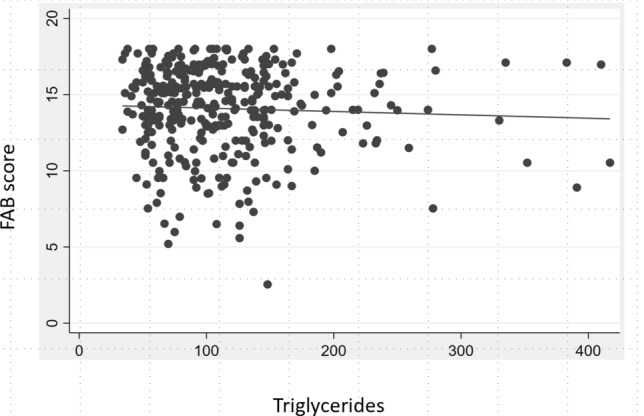
Negative correlation between Frontal Assessment Battery (FAB) score and Triglycerides (TG) serum levels in the woman group (*r* = − 0.226; *p* value 0.005)

Regarding the 148 women, at univariate analysis a positive association between hypertriglyceridemia and FAB score under cutoff was found (OR 3.4; 95%CI 1.29–9.03; *p* value 0.013). A slightly lower positive association, even if not statistically significant, was also found at multivariate analysis, adjusting for age, disease duration, education and UPDRS-ME score (Table 2). Moreover, always in women, a statistically significant negative correlation was found between FAB score and TG serum levels (*r* = − 0.226; *p* value 0.005) (Fig. 1). This correlation was also confirmed at the linear regression, after adjusting by age, education, disease duration and UPDRS-ME (coeff: − 0.009; 95%CI − 0.018 to − 0.001; *p* value 0.028). No other statistically significant correlations were found between FAB and other lipids in women.

Concerning the 200 men, a statistically significant negative association between hypercholesterolemia and FAB score under cutoff (OR 0.4; 95%CI 0.17–0.84; *p* value 0.018) and between high LDL and FAB score under cutoff (OR 0.4; 95%CI 0.18–0.90; *p* value 0.027) were found; at multivariate analysis, only the former association was confirmed (Table 2). Differently from women, among men no statistically significant correlations were found between serum lipid fractions and FAB score.

## Discussion

Sex-specific differences in serum lipid profile have been frequently reported in healthy aged individuals and associated with worse cognitive performance (Lu et al. 2017). Although it has been reported that cognitive abilities of PD patients can be influenced by sex (Nicoletti et al. 2017; Kurlawala et al. 2021), sex-specific mechanisms explaining the different role of specific lipid fractions on cognitive performance are still largely under-investigated. The present cross-sectional study assessed possible associations between serum lipid profile and cognitive performance in a large cohort of non-demented PD patients, exploring sex-specific differences.

In the whole PACOS cohort, none of the lipid fraction was found to be associated with executive dysfunction. This data is in agreement with the recent study performed by Choe et al. (2021) reporting no associations between lipid levels and cognitive functioning in a longitudinal study on advanced PD patients. Conversely, in a cross-sectional study on early PD patients, Huang et al. (2018) reported a strong association between higher serum TG levels and poorer executive and visuospatial functioning.

Consistently to previous findings (Sterling et al. 2016; Bakeberg et al. 2021), the women of the PACOS cohort showed higher TC, HDL and LDL serum levels than men. However, while Bekeberg et al. (2021) reported a strong association between high HDL levels and cognitive impairment (including attentive and executive functioning), in women with PD but not in men, in the PACOS cohort HDL was not associated with executive functioning neither in the whole sample, nor in the sample stratified by sex. Moreover, it should be noted that the study performed by Bekeberg et al. (2021) did not include the TG evaluation.

Interestingly, in the present study, sex-specific differences concerning the association between lipid fractions and cognitive abilities were found. In particular, only in women, a positive association between executive dysfunction and hypertriglyceridemia was found. Similarly, a negative correlation between triglycerides and FAB score was found only in women. On the contrary, among men, an inverse association was found between hypercholesterolemia and normal FAB performance. While in the general middle-older age population (Lu et al. 2017), high LDL levels have been associated with better performance in test exploring executive functioning, our results did not agree with the study performed by Bekeberg et al. (2021), reporting no associations between LDL and TC levels and cognitive performances in PD patients.

The neurobiological bases of the sex-specific different contribute of lipids fractions on cognitive performance in PD are still unclear, probably due to the few studies available to date which have carried out sex-stratified analysis. Elevated triglyceride levels might compromise the blood–brain-barrier transport of insulin and other hormones (Banks 2012) exerting a pro-inflammatory effect which may negatively influence cognitive performance. Previous studies have reported that high triglycerides levels exert a worse cardiovascular outcome in women than in men (Knopp et al. 2006). Similarly, it could be assumed that also in subjects with cognitive impairment, hypertriglyceridemia may exert a more detrimental effect in women than in men.

The role of cholesterol in cognition is even more controversial and fascinating. Hence, while in normal aging hypercholesterolemia has been associated with executive dysfunction, on the contrary in PD patients high LDL and TC levels have been associated with better executive functioning (Sterling et al. 2016). Moreover, considering that a previous study reported an association between hypercholesterolemia and slower clinical progression in men with PD (Huang et al. 2018), our findings raise the possibility that hypercholesterolemia may play a sex-specific, beneficial role in patients with PD.

Our study has several strengths, including the relatively large number of subjects included in the PACOS cohort and the homogeneous nature of the study group, which may reduce confounding. Nevertheless, some limitations should be mentioned. The hospital-based study design did not allow us to exclude a selection bias related to the possible presence of more severe cases. Nonetheless, the PACOS cohort is made up of PD patients with a short disease duration and a mild to moderate stage of disease as deduced by the mean Hoehn and Yahr stage. Moreover, the cross-sectional nature of the study did not allow us to evaluate the causality between lipid fractions and executive functioning. Furthermore, although we adjusted for major potential confounders, residual confounding (e.g. lipid lowering medications, physical exercise and diet) cannot be excluded given the observational design of the study. Finally, the lack of a healthy controls group did not allow us to conclude that the association between lipids fractions and executive functioning is specific of PD.

However, to the best of our knowledge, this is the largest study which have evaluated the specific sex-stratified role of serum lipid levels (LDL, HDL, TC and TG) on executive functioning carried out to date in PD patients. Since sex-specific differences are often disregarded and uncontrolled for, our study could contribute to identify sex-specific biomarker for cognitive decline in PD. Longitudinal studies carried out in the PACOS and other cohorts are needed to confirm and extend the present findings.

## Data Availability

Anonymized data will be made available on reasonable request.
